# Identification of novel mureidomycin analogues via rational activation of a cryptic gene cluster in *Streptomyces roseosporus* NRRL 15998

**DOI:** 10.1038/srep14111

**Published:** 2015-09-15

**Authors:** Lingjuan Jiang, Lu Wang, Jihui Zhang, Hao Liu, Bin Hong, Huarong Tan, Guoqing Niu

**Affiliations:** 1 State Key Laboratory of Microbial Resources, Institute of Microbiology, Chinese Academy of Sciences, Beijing, China; 2 University of Chinese Academy of Sciences, Beijing, China; 3 Key Laboratory of Industrial Fermentation Microbiology, Ministry of Education, College of Biotechnology, Tianjin University of Science and Technology, Tianjin, China; 4 Key Laboratory of Biotechnology of Antibiotics of Ministry of Health, Institute of Medicinal Biotechnology, Chinese Academy of Medical Sciences and Peking Union Medical College, Beijing, China

## Abstract

Antimicrobial agents are urgently needed to tackle the growing threat of antibiotic-resistant pathogens. An important source of new antimicrobials is the large repertoire of cryptic gene clusters embedded in microbial genomes. Genome mining revealed a napsamycin/mureidomycin biosynthetic gene cluster in the chromosome of *Streptomyces roseosporus* NRRL 15998. The cryptic gene cluster was activated by constitutive expression of a foreign activator gene *ssaA* from sansanmycin biosynthetic gene cluster of *Streptomyces* sp. strain SS. Expression of the gene cluster was verified by RT-PCR analysis of key biosynthetic genes. The activated metabolites demonstrated potent inhibitory activity against the highly refractory pathogen *Pseudomonas aeruginosa*, and characterization of the metabolites led to the discovery of eight acetylated mureidomycin analogues. To our surprise, constitutive expression of the native activator gene *SSGG_02995*, a *ssaA* homologue in *S. roseosporus* NRRL 15998, has no beneficial effect on mureidomycin stimulation. This study provides a new way to activate cryptic gene cluster for the acquisition of novel antibiotics and will accelerate the exploitation of prodigious natural products in *Streptomyces*.

Mureidomycins (mureidomycin A, B, C and D), initially isolated from *Streptomyces flavidovirens*^1^,^2^, belong to the uridyl peptide antibiotics which also include pacidamycins from *Streptomyces coeruleorubidus*^3^, napsamycins from *Streptomyces* sp. DSM 5940^4^, and sansanmycins from *Streptomyces* sp. strain SS^5^. The uridyl peptide antibiotics share a common structural scaffold, an unique 3′-deoxy-4′,5′-enamino-uridine nucleoside linked via an enamide bond to a pseudotetra- or pentapeptide backbone^6^. Mureidomycins are more closely related to napsamycins. The main difference between them is that mureidomycins contain *m*-tyrosine (*m*-Tyr), and napsamycins contain bicyclic 6-hydroxy-tetrahydro-isoquinoline carboxylic acid (Htia) in the N-terminal of the peptide skeleton (Fig. 1a). Acting as competitive inhibitors of bacterial phospho-N-acetylmuramyl-pentapeptide translocase (translocase I, annotated as MraY), mureidomycins exert potent inhibitory activity against the highly refractory pathogen *Pseudomonas aeruginosa*^7^. It is noteworthy that mureidomycin C is active against *P. aeruginosa* resistant to β-lactam antibiotics, and it is also effective in treating *P. aeruginosa* infection in mice^7^.

The gene clusters for napsamycin, pacidamycin and sansanmycin biosynthesis have been cloned^8^,^9^,^10^,^11^. Comparison of these gene clusters showed that organization of genes in clusters of napsamycin and sansanmycin has a striking resemblance, while the genetic organization of the pacidamycin gene cluster is different^12^. The napsamycin gene cluster was identified in *Streptomyces* sp. DSM 5940, and heterologous expression of the gene cluster in *Streptomyces coelicolor* M1154 led to the production of both napsamycins and mureidomycins, suggesting that they share the same biosynthetic pathway^8^. Genome mining revealed a similar gene cluster embedded in the chromosome of *Streptomyces roseosporus* NRRL 15998, and the gene cluster has been cloned from *S. roseosporus* NRRL 15998 via phage ϕBT1 integrase-mediated site-specific recombination^13^. The existence of the gene cluster indicated that *S. roseosporus* NRRL 15998 has the full biosynthetic capacity to produce napsamycins and/or mureidomycins. However, the production of napsamycins/mureidomycins or related compounds has not been reported. Therefore, the gene cluster of interest has been considered as silent in *S. roseosporus* NRRL 15998.

The alarming rise in the prevalence of antibiotic resistance poses a serious public health threat, and it has coincided with a dwindling supply of new antibiotics^6^. Sequencing of several *Streptomyces* genomes revealed the presence of a large number of cryptic secondary metabolites biosynthetic gene clusters, which represents an important source for the discovery of new antibiotics^14^. Currently, several methods have been generally used for the activation of cryptic gene clusters^15^. Among them, two most commonly used methods are heterologous expression of gene clusters in different host strains and genetic manipulation of cluster-situated regulator (CSR) genes. Genetic manipulation of CSR genes, simply to overexpress activators or delete repressors, is a straightforward and effective strategy^15^. A giant cryptic type I modular PKS gene cluster of *Streptomyces ambofaciens* ATCC 23877 was induced by overexpression of a Large ATP binding of the LuxR (LAL) family regulatory gene, leading to the discovery of four unusual glycosylated macrolides^16^. Similarly, expression of a cryptic gene cluster was triggered by the overexpression of a StrR-like regulatory gene, followed by identification of a type III glycopeptides^17^. Deletion of *scbR2* and its homologue *jadR2* activated the normally cryptic type I polyketide synthase gene cluster in *S. coelicolor* and jadomycin gene cluster *in Streptomyces venezuelae*^18^,^19^.

Here, we report the activation of a cryptic mureidomycin biosynthetic gene cluster and identification of eight acetylated mureidomycin analogues. The activation of mureidomycin production in *S. roseosporus* NRRL 15998 was achieved by constitutive expression of *ssaA* from *Streptomyces* sp. strain SS. It is surprising that constitutive expression of *SSGG_02995*, a *ssaA* homologue in *S. roseosporus* NRRL 15998, has no positive effect on mureidomycin production. This work provides the strategy to activate cryptic gene cluster by overexpression of a foreign activator gene, and will accelerate studies on the biosynthesis and pathway engineering of mureidomycins.

## Results

### Analysis of the napsamycin/mureidomycin biosynthetic gene cluster in *S. roseosporus* NRRL 15998

**Figure 1:**
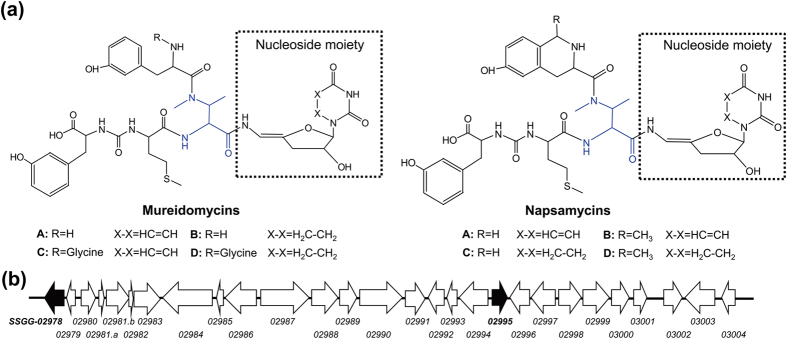
Structures of napsamycins/mureidomycins and organization of their putative biosynthetic gene cluster in *S. roseosporus* NRRL 15998. (**a**) Structures of napsamycins and mureidomycins. The unique nucleoside moiety was surrounded by a dashed rectangle and the featured N-methyl-2,3-diaminobutyric acid (N-methyl-DABA) was shaded in blue. (**b**) Organization of the mureidomycin biosynthetic gene cluster in *S. roseosporus* NRRL 15998. The gene cluster contains 28 *orfs*, and the two putative regulatory genes (*SSGG_02978* and *SSGG_02995*) are shaded in black.

Genome mining revealed a cryptic napsamycin/mureidomycin biosynthetic gene cluster in the supercont 3.1 genomic scaffold of *S. roseosporus* NRRL 15998 (GenBank accession number DS999644.1). The FramePlot 4.0 beta online program showed that the cluster consists of 28 complete open reading frames (ORFs) (Fig. 1b), and BLAST homology searches revealed high similarities (94–99% identity) for all 28 deduced proteins with corresponding proteins encoded by genes within the napsamycin gene cluster of *Streptomyces* sp. DSM 5940 (Supplementary Table S1). It should be noted that there are two ORFs (*SSGG_02981.a* and *SSGG_02981.b*) in the annotated *SSGG_02981* (Supplementary Fig. S1 and Table S1), and further examination of the gene cluster identified two regulatory genes, *SSGG_02978* and *SSGG_02995* (Fig. 1b and Supplementary Table S1). Sequence analysis showed that *SSGG_02978* encodes a putative ArsR family transcriptional regulator. SSGG02978 shows 99% identity with NpsA (GenBank accession number ADY76657.1) from *Streptomyces* sp. DSM 5940 and 47% identity with Y19_18380 (WP_017239216.1) from *Streptomyces* sp. strain SS. Though NpsA was proposed to be a transcriptional repressor^8^, functions of both NpsA and Y19_18380 remain unknown. Based on results of the FramePlot program and BLAST analysis, the annotation of *SSGG_02995* should be revised and the amended nucleotide sequence was provided (Supplementary Fig. S1). *SSGG_02995* encodes a hypothetical protein SSGG02995 (ZP_04694256.1) showing 97% identity with NpsM (ADY76675.1) from *Streptomyces* sp. DSM 5940, 84% identity with SsaA (AGG82463.1) from *Streptomyces* sp. strain SS, and 81% identity with PacA (ADN26237.1) from *S. coeruleorubidus* (Supplementary Fig. S2). SsaA was characterized as an activator of sansanmycin production in *Streptomyces* sp. strain SS^9^, while functions of NpsM and PacA have not been investigated.

### Effect of *SSGG_02978* deletion on mureidomycin production

**Figure 2:**
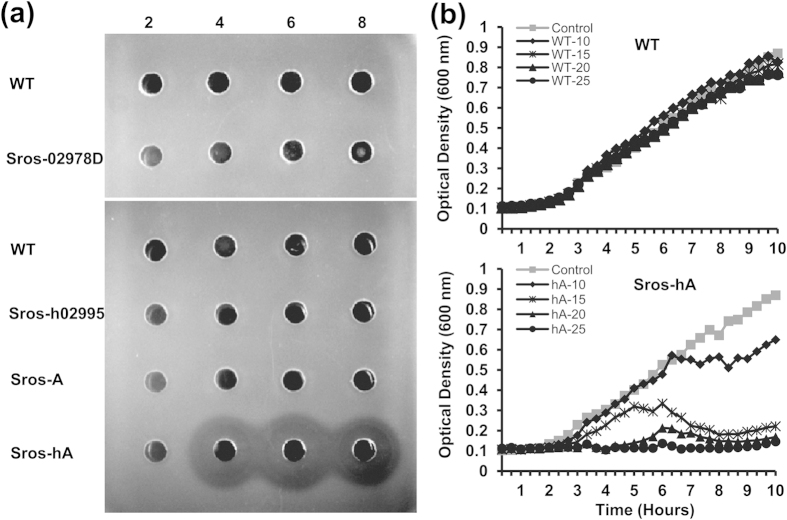
Effect of mureidomycin production on the growth of *P. aeruginosa* PA14. (**a**) Bioassay of mureidomycin production in *S. roseosporus* NRRL 15998 (WT) and its derivatives. The strains were cultured in liquid ISP-2 for 2-8 days, and 100 μl of supernatants from culture broths were assayed for bioactivity against *P. aeruginosa* PA14. (**b**) The growth curves of *P. aeruginosa* PA14 are shown when cultured in the presence of fermentation extract from WT or Sros-hA (hA). Shown here are the average optical density values of three replicate wells for each time point. This experiment was performed three times with similar results. Wells containing different volumes (10, 15, 20, and 25 μl) of extract from either WT or Sros-hA were indicated. ISP-2 medium serves as negative controls.

**Figure 3:**
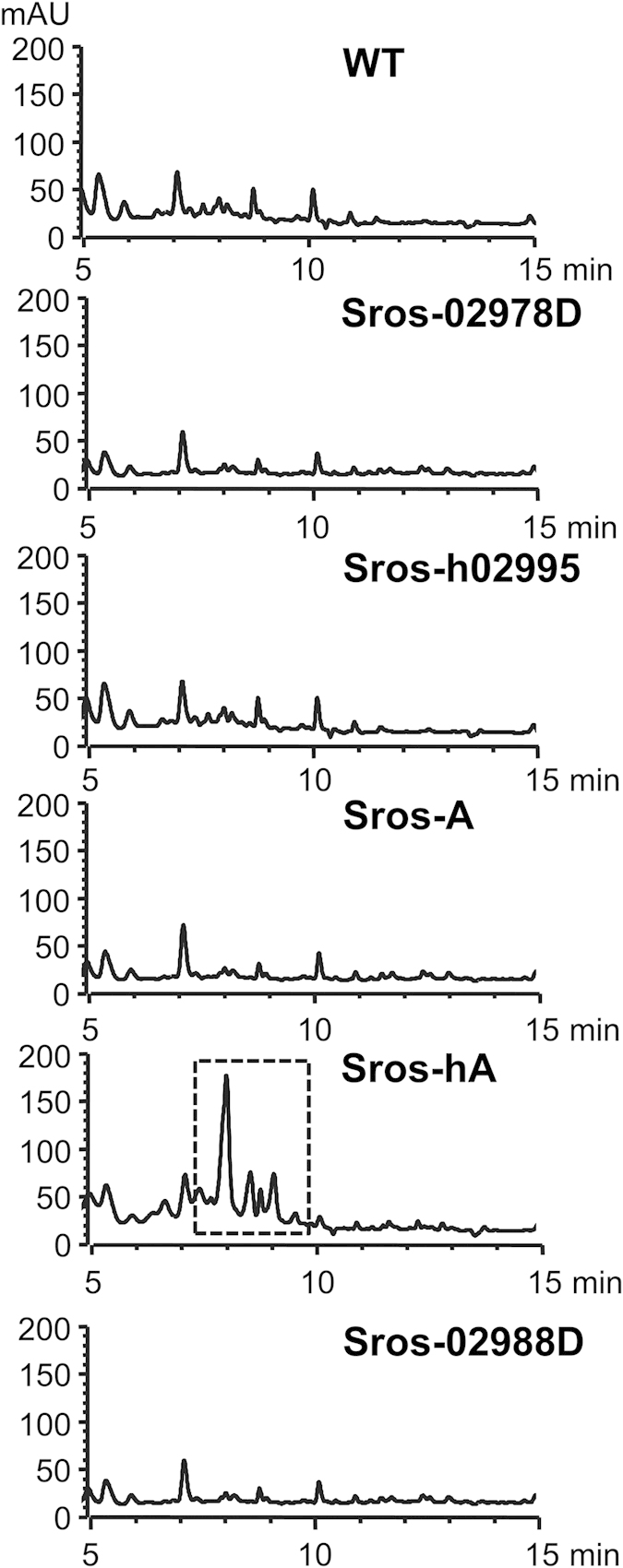
HPLC analysis of fermentation extracts from *S. roseosporus* NRRL 15998 and its derivatives. The fraction with distinct peaks only detected in the Sros-hA was surrounded by a dashed rectangle.

Initially, we chose to use heterologous expression for activation of the cryptic napsamycin/mureidomycin biosynthetic gene cluster. The gene cluster cloned from *S. roseosporus* NRRL 15998 was transferred into several well-developed surrogate hosts including *Streptomyces albus* J1074, *S. coelicolor* M1146, *S. coelicolor* M1152 and *S. coelicolor* M1154. However, no inhibitory activity against *P. aeruginosa* was observed with these recombinant strains containing the gene cluster (data not shown). Our strategy was then redirected to genetic manipulation of the two CSRs within the gene cluster. Since ArsR family transcriptional regulators typically serve as repressors^15^, a *SSGG_02978* disruption mutant (Sros-02978D) was constructed and tested for antimicrobial activity against *P. aeruginosa* PA14 and *P. aeruginosa* PAO1. Like the wild-type strain (WT), no inhibition zones were observed with Sros-02978D (Fig. 2a and Supplementary Fig. S3). High-performance liquid chromatography (HPLC) profile of culture filtrate from Sros-02978D showed no difference with that of culture filtrate from the WT strain (Fig. 3). These results showed that deletion of *SSGG_02978* failed to activate the cryptic gene cluster in *S. roseosporus* NRRL 15998.

### Activation of the cryptic napsamycin/mureidomycin biosynthetic gene cluster

Next, we determined to test whether overexpression of *SSGG_02995* could activate the cryptic gene cluster. To do that, *SSGG_02995* was cloned into pSET152 under the control of the constitutive *hrdB* promoter and the resulting recombinant plasmid pSET152::h02995 was transferred into the WT strain. Culture filtrate from the recombinant strain Sros-h02995 still could not inhibit the growth of *P. aeruginosa* PA14 (Fig. 2a). We then sought to introduce its homologue *ssaA* from *Streptomyces* sp. strain SS into the WT strain. For this purpose, *ssaA* was inserted into pSET152 under the control of its natural promoter and the *hrdB* promoter, and these constructs (pSET152::A and pSET152::hA) were introduced into the WT strain respectively to obtain Sros-A and Sros-hA. It should be noted that the annotation of *ssaA* was revised based on results of FramePlot program and BLAST analysis, and the amended *ssaA* coding sequence was provided (Supplementary Fig. S1). Both Sros-A and Sros-hA were subjected to antimicrobial assays. Sros-A could not inhibit the growth of *P. aeruginosa* PA14, while Sros-hA showed distinct inhibition zones against *P. aeruginosa* PA14 and *P. aeruginosa* PAO1 (Fig. 2a and Supplementary Fig. S3). Next, we compared the growth characteristics of *P. aeruginosa* PA14 in the presence of fermentation extract from the WT or Sros-hA. When grown in LBNS media containing fermentation extract from the WT, growth patterns of *P. aeruginosa* PA14 was identical to that of *P. aeruginosa* PA14 grown in LBNS media supplemented with ISP-2 controls. However, *P. aeruginosa* PA14 exhibited a marked reduction in growth rate with supplementation of fermentation extract from the Sros-hA, and a complete inhibition of growth was observed when 1/10 volume of fermentation extracts were added (Fig. 2b). Moreover, HPLC analysis revealed distinct peaks in the culture filtrate of Sros-hA that were absent in the culture filtrates from the WT, Sros-02978D, Sros-h02995 and Sros-A (Fig. 3).

To confirm the metabolites were the products of the gene cluster, we chose to inactivate the *SSGG_02988* in Sros-hA. *SSGG_02988* is a homologue of *npsG* from *Streptomyces* sp. DSM 5940, which was proposed to be involved in the biosynthesis of the unusual 2,3-diaminobutyric acid (DABA)^8^. The resulting *SSGG_02988* mutants (Sros-02988D) were subjected to antimicrobial assays. Unlike Sros-hA, culture filtrates from Sros-02988D lost the ability to inhibit the growth of *P. aeruginosa* PA14 (Supplementary Fig. S4). Furthermore, HPLC analysis showed that those distinct peaks observed in the culture filtrate of Sros-hA were absent in the culture filtrate from Sros-02988D (Fig. 3). These results demonstrated that the gene cluster is responsible for the production of anti-*Pseudomonas* metabolites in Sros-hA.

**Figure 4:**
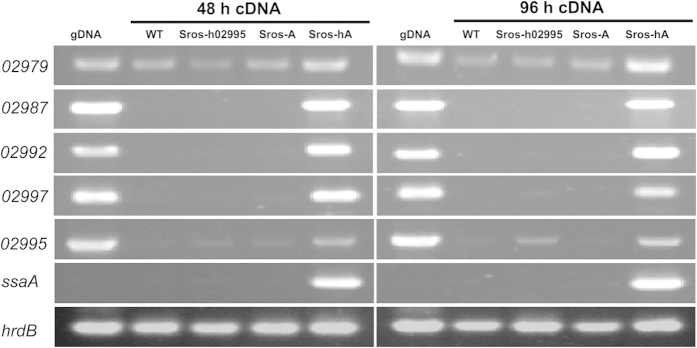
Transcriptional analysis of representative genes by semi-quantitative RT-PCR. Total RNAs were isolated from mycelia of the wild-type strain (WT), Sros-h02995, Sros-A and Sros-hA after fermentation for 48 h and 96 h. The constitutive *hrdB* transcript was used as an internal control. Twenty-eight cycles of PCR were routinely employed.

Previous study showed that SsaA can bind to five different promoter regions of the sansanmycin biosynthetic gene cluster and a consensus SsaA binding sequence was identified^9^. Interestingly, the *S. roseosporus* NRRL 15998 napsamycin/mureidomycin gene cluster also contains the highly conserved SsaA binding sites in five different promoter regions^9^. To examine the effect of *ssaA* overexpression on biosynthetic genes, transcriptional analysis was performed by using semi-quantitative RT-PCR. The results showed that three key structural genes (*SSGG_02987*, *SSGG_02992* and *SSGG_02997*) were expressed only in Sros-hA, while the expression of *SSGG_02979* was detected in all four strains with significantly increased transcription level in Sros-hA (Fig. 4), suggesting that the foreign *ssaA* from *Streptomyces* sp. strain SS can function as a pathway-specific activator gene in *S. roseosporus* NRRL 15998. Furthermore, we also evaluated the expression of *SSGG_02995* and *ssaA*. Transcription of *SSGG_02995* was detected both in Sros-h02995 and Sros-hA, while transcription of *ssaA* was detected only in Sros-hA. From these results, we concluded that *S. roseosporus* NRRL 15998 has indeed the genetic capacity to produce napsamycin/mureidomycin or related analogues, and these antimicrobial compounds are only detected after transcriptional activation with *ssaA* from *Streptomyces* sp. strain SS.

### Identification of mureidomycin analogues

**Figure 5:**
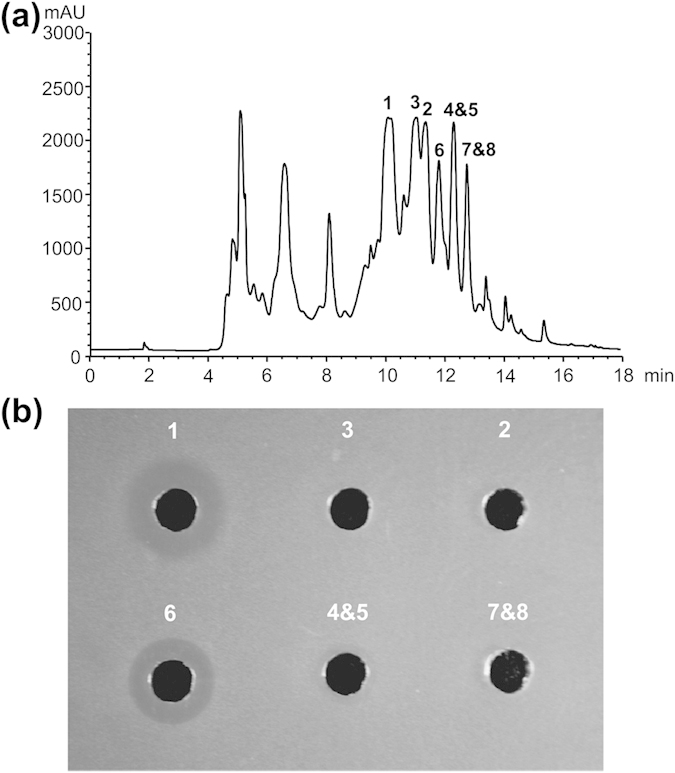
HPLC analysis of six fractions containing different components of mureidomycins (a) and bioassay of each fraction against *P. aeruginosa* PA14 (b). (1) fraction containing compound **1** (*N*-acetylmureidomycin E); (2) fraction containing compound **2** (*N*-acetylmureidomycin A); (3) fraction containing compound **3** (*N*-acetylmureidomycin A); (4 & 5) fraction containing compound **4** (*N*-acetylmureidomycin F) and **5** (*N*-acetylmureidomycin G); (6) fraction containing compound **6** (*N*-acetylmureidomycin H); (7 & 8) fraction containing compound **7** (*N*-acetylmureidomycin I) and **8** (*N*-acetylmureidomycin J).

To identify the products of the activated gene cluster, we first compared the metabolic profiles of the WT and Sros-hA, and characterized eight compounds present only in the extract of Sros-hA with [M+H]^+^ ions at *m/z* of 899.2, 885.3, 883.3, 869.3, 867.3, 865.3, 851.3 and 849.3 by ESI-MS. Then, we optimized the isolation procedure to separate those distinct peaks in the culture filtrate of Sros-hA into six fractions, and eight compounds (compound **1**–**8**) corresponding to those ions were identified by ESI-MS and MS/MS analysis (Fig. 5a). In the meantime, fractions containing different compounds were also subjected to bioassay against *P. aeruginosa* PA14. Fractions containing compound **1** or **6** showed distinct inhibition zones against *P. aeruginosa* PA14 and fraction containing compound **4** & **5** showed minor inhibition zones against *P. aeruginosa* PA14, while no inhibition zones were found with the rest fractions (Fig. 5b). Of these eight compounds, compound **1** and **3** were selected for NMR analysis. The detailed analyses of spectrometric data were as follows.

**Figure 6:**
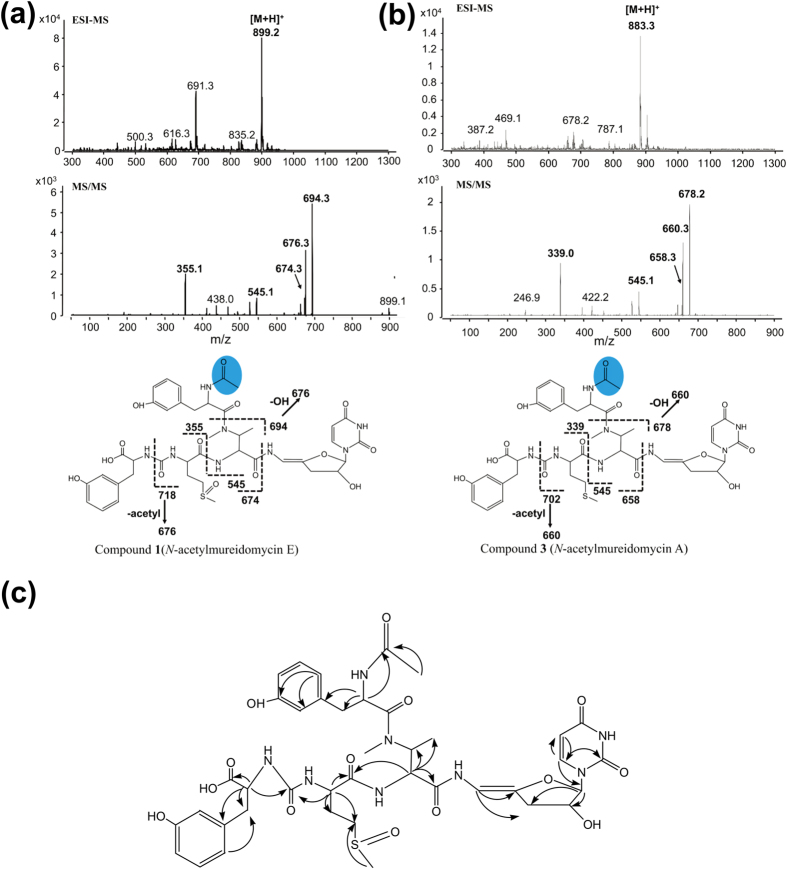
Identification of compound 1 (*N*-acetylmureidomycin E) and compound 3 (*N*-acetylmureidomycin A). (**a**) ESI-MS and MS/MS analyses of *N*-acetylmureidomycin E (**b**) ESI-MS and MS/MS analyses of *N*-acetylmureidomycin A. The unique acetyl group was shadowed with an oval. (**c**) Structure of *N*-acetylmureidomycin E. HMBC was used for structure determination.

The ESI-MS spectrum of compound **1** displayed the [M+H]^+^ ion at *m/z* 899.2. MS/MS analysis showed that most fragments of compound **1** were the same as those of mureidomycin A^2^, except for two fragments at *m/z* 694 and 355 (Fig. 6a). These two fragments are respectively 16 Da larger than the two daughter ions (*m/z* 678 and 339) of mureidomycin A, suggesting the methionine (Met) of mureidomycin A was replaced by a methionine sulfoxide (Met^SO^) in compound **1** (Fig. 6a). Next, the purified compound **1** was subjected to NMR analysis (Supplementary Fig. S8-S11) and the assignments of ^1^H-NMR and ^13^C-NMR signals were listed in Table 1. Compared with ^1^H-NMR and ^13^C-NMR spectra of mureidomycin A, most signals of moieties *m*-Tyr, sugar, uracil and 2-amino-3-methylaminobutyric acid (AMBA) in compound **1** were consistent with those in mureidomycin A. The methyl proton signal (−SCH_3_) of mureidomycin A was shifted to low field (δ_H_ 2.54 ppm) in compound **1**, and δ_H_ 2.54 ppm was correlated to the carbon signal (δ_C_ 48.9 ppm) of Met^SO^-4 position. The extra signals δ_H_ 1.76 ppm (3H, s-, COCH_3_) and carbonyl carbon (δ_C_ 173.41 ppm) of compound **1** indicated the presence of an acetyl group. According to COSY and HMBC data, both -CH_3_ (δ_H_ 1.76 ppm) and -CH (δ_H_ 4.79 ppm, N-terminal *m*-Tyr-2) showed correlations to carbonyl carbon (δ_C_ 173.41 ppm) of the acetyl group (Supplementary Fig. S9-S11). Furthermore, HR-MS analysis showed the molecular formula of compound **1** to be C_40_H_51_N_8_O_14_S (*m/z* calculated: 899.324, found: 899.3236[M]^+^). Taken together, compound **1** is a N-terminal acetylated mureidomycin with Met^SO^ instead of Met in the peptide backbone (Fig. 6c). Thus, compound **1** was identified as a new compound and named *N*-acetylmureidomycin E.

The *m/z* of compound **2** (885.3 [M+H]^+^) is 42 larger than that of mureidomycin B (843.3 [M+H]^+^) and the fragmentation pattern (Supplementary Fig. S5a) is identical to that of *N*-acetylmureidomycin B^12^. Compound **2** was therefore identified as *N*-acetylmureidomycin B. Similarly, compound **3** (883.3 [M+H]^+^) (Fig. 6b) corresponds to mureidomycin A (841.3 [M+H]^+^)^8^ with the attachment of an acetyl group. This was further confirmed by comparative analysis of the MS/MS spectra of compound **3** (Fig. 6b) and *N*-acetylmureidomycin B^12^. Purified compound **3** was also subjected to NMR analysis (Supplementary Fig. S12). The data on ^1^H-NMR and ^13^C-NMR of compound **3** are similar to those of compound **1** except for *δ*_H_ 2.54 ppm at Met^SO^-6 position, which was shifted to high field 1.96 ppm in compound **3**, suggesting the presence of Met instead of Met^SO^. Furthermore, HR-MS analysis showed the molecular formula of compound 3 to be C_40_H_51_N_8_O_13_S (*m/z* calculated: 883.3291[M]^+^, found: 883.3290). Thus, compound **3** was identified as a new compound and named *N*-acetylmureidomycin A.

The *m/z* of compound **4** (869.3 [M+H]^+^) is 16 lower than *N*-acetylmureidomycin B (885.3 [M+H]^+^). Comparative analysis of the MS/MS spectra of compound **4** and *N*-acetylmureidomycin B indicated that compound **4** corresponds to *N*-acetylmureidomycin B lacking a hydroxyl group in the C-terminal *m*-Tyr, suggesting the C-terminal amino acid in compound **4** is a phenylalanine (Phe) (Supplementary Fig. S6a). Similarly, compound **5** (867.3 [M+H]^+^) corresponds to *N*-acetylmureidomycin A (883.3 [M+H]^+^) lacking a hydroxyl group in the C-terminal *m*-Tyr, suggesting the C-terminal amino acid in compound **5** is a Phe (Supplementary Fig. S6b). Thus, compound **4** and **5** are new compounds and named *N*-acetylmureidomycin F and G, respectively.

The *m/z* of compound **6** (865.3[M+H]^+^) is 18 lower than that of *N*-acetylmureidomycin A (883.3 [M+H]^+^). Comparative analysis of the MS/MS spectra of compound **6** and *N*-acetylmureidomycin A indicated that the loss of 18 in the position of Met, suggesting the presence of a leucine (Leu) or isoleucine (Ile) instead of Met in compound **6** (Supplementary Fig. S5b). Thus, compound **6** is a new compound and named *N*-acetylmureidomycin H.

The *m/z* of compound **7** (849.3 [M+H]^+^) is 16 lower than *N*-acetylmureidomycin H (865.3[M+H]^+^). Comparative analysis of the MS/MS spectra of compound **7** and *N*-acetylmureidomycin H indicated that compound **7** corresponds to *N*-acetylmureidomycin H lacking a hydroxyl group in the C-terminal *m*-Tyr, suggesting the C-terminal amino acid in compound **7** is a Phe (Supplementary Fig. S7a). Similarly, the *m/z* of compound **8** (851.3 [M+H]^+^) is 2 larger than that of compound **7,** and comparative analysis of the MS/MS spectra indicated that the compound contained a dihydrouracil instead of uracil in its nucleoside moiety (Supplementary Fig. S7b). Thus, compound **7** and **8** are new compounds and named *N*-acetylmureidomycin I and J, respectively.

**Figure 7:**
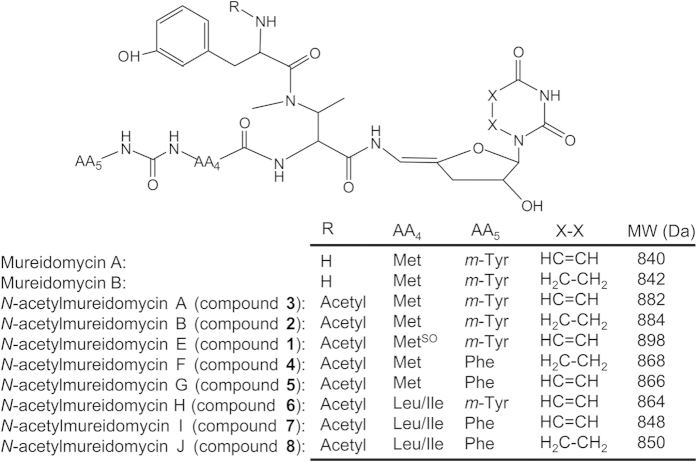
Comparison of mureidomycins with the analogues generated in this study. Met^SO^, methionine sulfoxide; MW: calculated molecular weight; Da: dalton.

In summary, we have identified eight mureidomycin analogues with acetylation at the N-terminal *m*-Tyr. Apart from the *N*-acetylmureidomycin A and B, other mureidomycin analogues also differ from the known mureidomycin A and B at AA_4_ and/or AA_5_ (Fig. 7). Both Met^SO^ and Leu have been identified at AA_4_ of sansanmycins, and Phe was previously identified only at AA_5_ of pacidamycins^9^,^11^. In this study, we observed the presence of these amino acids at AA_4_ and/or AA_5_ in six of the eight mureidomycin analogues (Fig. 7). Analogues generated by substitution of Met with Met^SO^ or Leu/Ile at AA_4_ displayed better anti-*Pseudomonas* activity than that of *N*-acetylmureidomycin A and B, while analogues with substitutions at AA_5_ displayed similar anti-*Pseudomonas* activity as *N*-acetylmureidomycin A and B (Fig. 5b). Generation of these analogues expanded the chemical diversity of mureidomycins and altered their biological activity accordingly. It also suggested that the non-ribosomal peptide synthetases (NRPSs) responsible for the selection and assembly of amino acid residues at AA_4_ and AA_5_ have a high degree of flexibility.

## Discussion

The uridyl peptide antibiotics consist of mureidomycin, napsamycin, pacidamycin and sansanmycin. Acting as MraY inhibitors, they are promising compounds for the development of new anti-*Pseudomonas* and anti-mycobacterial agents^6^. Comparison of the gene clusters for the biosynthesis of these uridyl peptide antibiotics showed that organization of genes in cluster of napsamycin/mureidomycin and sansanmycin has a striking resemblance, while the genetic organization of the pacidamycin gene cluster is different^12^. It is noteworthy that there are two putative regulatory genes (*npsA* and *npsM*) in napsamycin gene cluster, while only one regulatory gene (*pacA* or *ssaA*) was found in pacidamycin or sansanmycin gene cluster. Among them, only *ssaA* was investigated and characterized as an activator gene for sansanmycin production in *Streptomyces* sp. strain SS^9^. Base on the fact that NpsA and PacA are highly homologous to SsaA (Supplementary Fig. S2), *npsA* and *pacA* are predicted to function as activator gene in the biosynthesis of napsamycin and pacidamycin, respectively. However, function of *npsM* remains unknown. In this study, we deleted *SSGG_02978*, a *npsM* homologue, in *S. roseosporus* NRRL 15998. Analysis of the deletion mutant revealed that *SSGG_02978* has no effect on mureidomycin production. It seems that *SSGG_02978* or *npsM* is not involved in mureidomycin/napsamycin production.

For activation of cryptic gene clusters, a simple strategy is to overexpress or delete cluster-situated regulators (CSRs) encoded by genes within the gene cluster of interest. If the activator gene is not transcribed under routine laboratory conditions, activation of the cryptic gene cluster can be achieved simply by constitutive expression of the activator gene^16^. In our case, the activator gene *SSGG_02995* is transcribed at a fairly low level in the WT strain and it is not functional even after its overexpression in Sros-h02995 (Figs 2a and 4). Under these circumstances, it is necessary to evaluate activators from other gene clusters, especially those required for the biosynthesis of structurally similar antibiotics. Sometimes, it is even more complicated. Activation of cryptic gene cluster requires expression of a functional pleiotropic or global regulator. Activation of secondary metabolites produced by a cryptic gene cluster in *Streptomyces calvus* requires expression of a functional copy of *bldA*^20^, which encode the only tRNA capable of translating the leucine codon UUA.

We have successfully activated a silent mureidomycin biosynthetic gene cluster in *S. roseosporus* NRRL 15998 by constitutive expression of *ssaA* from *Streptomyces* sp. strain SS. Surprisingly, constitutive expression of *SSGG_02995*, a native *ssaA* homologue, failed to activate mureidomycin production in *S. roseosporus* NRRL 15998 (Fig. 2a). SSGG02995 and its orthologues contain an N-terminal fork head-associated (FHA) domain and a C-terminal LuxR-type helix-turn-helix (HTH) motif. Further examination of SSGG02995 in *S. roseosporus* NRRL 15998 showed that variations were mainly found in the FHA domain of SSGG02995, while the DBD domain remains essentially the same (Supplementary Fig. S2). It is plausible that SSGG02995 lost the ability to activate mureidomycin production due to amino acid substitutions in its FHA domain. This may explain why the mureidomycin biosynthetic gene cluster remains silent in several well-developed surrogate hosts. However, the regulatory mechanism of the aberrant SSGG02995 remains unknown.

We have identified eight acetylated mureidomycin analogues. Previous study suggested that NpsB, an N-acetyltransferase, is responsible for the formation of *N*-acetylmureidomycin B from mureidomycin B and acetyl-CoA^12^. In this study, the expression of *SSGG_02979*, a *npsB* homologue in *S. roseosporus* NRRL 15998, was increased significantly in Sros-hA. We therefore speculated that SSGG_02979 is responsible for the accumulation of acetylated mureidomycin analogues in *S. roseosporus* NRRL 15998. The N-acetylation reaction was proposed to contribute to a self-resistance of napsamycin in the producer strain^12^, and similar N-acetyltransferase-mediated reaction was reported to be responsible for self-resistance to the antimicrobial peptide edeine of *Brevibacillus brevis* Vm4^21^. However, the role of acetylation in mureidomycin self-resistance remains elusive until further experimental validation. Moreover, the substrate promiscuity of NRPSs was observed with variations at AA_4_ and/or AA_5_ of mureidomycin (Fig. 7). Similar observations were also found at different amino acid positions in the biosynthesis of pacidamycin and sansanmycin^22^,^23^. The substrate promiscuity of NRPSs is responsible for the diversity of these uridyl peptide antibiotics. It can also be explored for the generation of novel analogues by precursor-directed biosynthesis^22^,^23^. With the activation of mureidomycin biosynthetic gene cluster in *S. roseosporus* NRRL 15998, the same strategy could be used for generation of novel mureidomycin analogues, and systematic structure–activity relationship (SAR) studies can be carried out to evaluate their biological activities thereafter. If necessary, the NRPSs may be engineered to have a preference for amino acid substrates which can be incorporated to produce mureidomycin analogues with improved anti-*Pseudomonas* activity.

## Methods

### Bacterial strains, plasmids and primers

Bacterial strains and plasmids used in this study are listed in Supplementary Table S2, and primers are listed in Supplementary Table S3. *S. roseosporus* NRRL 15998 is the wild-type strain used for activation of the cryptic mureidomycin biosynthetic gene cluster. *S. albus* J1074, *S. coelicolor* M1146, *S. coelicolor* M1152 and *S. coelicolor* M1154 serve as hosts for heterologous expression of the cryptic mureidomycin biosynthetic gene cluster. *P. aeruginosa* PAO1 and *P. aeruginosa* PA14 were used as indicator strains for mureidomycin bioassay. *Escherichia coli* Top10 was used as a general host for propagating plasmids. *E. coli* ET12567 (pUZ8002) was used as a host for transferring DNA from *E. coli* to *Streptomyces* by intergeneric conjugation. *S. roseosporus* NRRL 15998 was cultured at 28 °C on AS-1 agar medium or in tryptic soy broth (TSB) liquid medium^24^. *P. aeruginosa* was grown at 37 °C in Luria–Bertani lacking sodium chloride (LBNS)^25^. General approaches for *E. coli* or *Streptomyces* manipulations were performed according to standard procedures^26^,^27^.

### Sequence analysis

The nucleotide sequence of the putative napsamycin/mureidomycin biosynthetic gene cluster is available in the GenBank database under accession number DS999644.1. The ORFs were deduced from the sequence with FramePlot 4.0 beta program (http://nocardia.nih.go.jp/fp4). The corresponding deduced proteins were compared with proteins in the databases by available BLAST methods (http://www.ncbi.nlm.nih.gov/blast/) or ClustalW2 program (http://www.ebi.ac.uk/Tools/msa/clustalw2/). The program HHpred was used for protein structure prediction (http://toolkit.tuebingen.mpg.de/hhpred/).

### Construction of Sros-h02995, Sros-A and Sros-hA

*SSGG_02995* coding region and the *hrdB* promoter were amplified respectively with primer pair 02995-F/R and hrdBp-F/R from genomic DNA of *S. roseosporus* NRRL 15998 and *Streptomyces coelicolor* M145. Prior to polymerase chain reaction (PCR) amplification, hrdBp-R was phosphorylated with T4 polynucleotide kinase to facilitate subsequent ligation reactions. The coding region of *SSGG_02995* was digested with *Not*I, and the *hrdB* promoter was digested with *Xba*I. The two fragments were ligated together with *Xba*I/*Not*I digested pSET152 in a three-piece ligation reaction to generate pSET152::h02995. The plasmid pSET152::hA was constructed in a similar way except that primers ssaAorf-F and ssaA-R were used for amplification of *ssaA* coding region from cosmid 13R-1 9. For the construction of pSET152::A, a fragment covering the coding region of *ssaA* and its upstream region was amplified from cosmid 13R-1 by using primers ssaA-F and sssA-R. The amplified fragment was digested with *Xba*I and *Not*I and then inserted into the corresponding sites of pSET152 to generate pSET152::A. The plasmids pSET152::h02995, pSET152::A and pSET152::hA were introduced respectively into *S. roseosporus* NRRL 15998 to obtain recombinant strains, Sros-h02995, Sros-A and Sros-hA.

### Construction of mutants

*SSGG_02978* deletion mutant (Sros-02978D) in *S. roseosporus* NRRL 15998 was constructed via double-crossover homologous recombination. To generate the construct, two 2.0 kb fragments flanking *SSGG_02978* were amplified using primer pairs 02978-UpF/UpR and 02978-DnF/DnR with genomic DNA from *S. roseosporus* NRRL 15998 as template. The two fragments were digested with *Xba*I/*Spe*I and *Spe*I/*Eco*RV, and ligated into *Xba*I/*Eco*RV digested pKC1139 to obtain pKC1139::02978UD. The kanamycin resistance gene (*neo*) was amplified from pUC119::*neo*^28^ by using primers Kan-F and Kan-R. The resistance cassette was digested with *Spe*I and inserted into the *Spe*I linearized pKC1139::02978UD. The resulting pKC1139::02978UDneo was then transferred into *S. roseosporus* NRRL 15998 by intergeneric conjugation^27^. Spores of transformants were harvested and spread on AS-1 agar plates containing kanamycin. After growing at 40 °C for 4 days, colonies were replicated on AS-1 agar plates containing kanamycin or apramycin. Double-crossover exconjugants are apramycin sensitive (Apr^s^) and kanamycin resistant (Kan^r^). Apr^s^/Kan^r^ strains were then verified by PCR analysis.

For the construction of *SSGG_02988* disruption mutant in Sros-hA, approximately 500 bp internal fragments of *SSGG_02988* were amplified using primer pair 02988-SCF/SCR with genomic DNA from *S. roseosporus* NRRL 15998 as template. The amplified fragment was inserted into *Eco*RV site of pKC1139hph, which is a derivative of pKC1139 with the replacement of apramycin resistance gene by hygromycin resistance gene. The resulting pKC1139hph::02988SC was then introduced into Sros-hA by intergeneric conjugation^27^. Spores of transformants were collected and spread on AS-1 agar plates containing hygromycin. After growing at 40 °C for 4 days, colonies were patched on AS-1 agar plates containing hygromycin and then verified by PCR analysis.

### RNA extraction and semi-quantitative RT-PCR

Total RNAs were isolated from cultures of the wild-type (WT) strain, Sros-h02995, Sros-A and Sros-hA grown in ISP-2 media at 48 and 96 h as described previously^29^. The RNA samples were treated using RQ1 RNase-free DNase (Promega) to remove genomic DNA. Synthesis of cDNA was the same as described previously^30^. For semi-quantitative RT-PCR analysis, the reaction conditions were as follows: 95 °C for 5 min, followed by amplification cycles consisting of 45 s denaturation at 95 °C, 30 s annealing at 60 °C to 62 °C (depending on the set of primers used), and 40 s extension at 72 °C, and a final extension of 8 min at 72 °C. Products were detected by 1.5% agarose gel electrophoresis and visualized by staining with ethidium bromide.

### Production and analysis of mureidomycins

*S. roseosporus* and its derivatives were inoculated in 10 ml liquid TSB and cultured for 36 h as seed culture, then 1 ml of seed culture was transferred into a shake flask containing 100 ml ISP-2 medium (4 g l^−1^yeast extract, 10 g l^−1^ malt extract, 4 g l^−1^ glucose). The cultures were incubated at 28°C for different days before fermentation broths were collected by centrifugation. The supernatants were filtered through a Millipore membrane (pore diameter, 0.22 μm) and 10 μl of sample was used for HPLC analysis. Separation of mureidomycins was achieved with an Agilent 1100 HPLC system and a ZORBAX SB-C18 column (5 μm, 4.6 × 250 mm). HPLC conditions were as follows: gradient elution with buffer A (0.1% [vol/vol] methanoic acid in acetonitrile) and buffer B (0.1% [vol/vol] methanoic acid in ddH_2_O), flow rate at 1.0 ml/min, ultraviolet detection at wavelength of 260 nm. The elution profile was a hold at 20% buffer A over 2 min, a linear gradient of 20%–100% buffer A over 20 min, a hold at 100% buffer A over 6 min, a linear gradient of 100%–20% buffer A over 2 min and a final hold at 20% buffer A over 5 min.

### Bioassays of mureidomycins

Bioassay of mureidomycins against *P. aeruginosa* was performed as follows. Cultures of indicator strains (1 ml overnight culture of *P. aeruginosa* PAO1 or *P. aeruginosa* PA14) were well dispersed in 100 ml pre-dissolved LBNS agar and poured into a 15 cm plate. Supernatants of 100 μl were added into holes with diameter of 7 mm on the plates. For preparation of agar plugs, *S. roseosporus* and its derivatives were patched on ISP-2 agar. After incubation at 28 °C for 2–8 days, agar plugs were prepared from the patches and placed on the surface of bioassay plates. The zone of inhibition was assessed after overnight incubation at 37 °C.

For measuring the growth curves of *P. aeruginosa* PA14, the test strains were first grown overnight in LBNS medium. The overnight cultures were then diluted to 0.5% in LBNS containing different volumes (0, 10, 15, 20, 25 μl) of fermentation extracts from the WT or Sros-hA. A total 200 μl of cell suspension was added to each well of 100-well microplate, and the plate was then placed inside a Boscreen C automated system. Optical density at a wavelength of 600 nm was measured at regular intervals of 20 min over a 10 h period. Wells containing LBNS with ISP-2 medium were used as negative controls.

### Isolation and identification of mureidomycins

Seed culture of Sros-hA was prepared in 100 ml liquid TSB and transferred into 10 L ISP-2 medium. After fermentation for 4 days, the culture broth was passed through filter paper to remove the mycelia. The sample was chromatographed on a macroporous absorption resin HP-20 column (Mitsubishi), washed consecutively with 3 L of water and 3 L of 10% ethanol, and then eluted with 5 L of 25% ethanol. The eluate was concentrated to a small volume *in vacuo* and subjected to a Sephadex LH-20 column. The column was eluted with 10% methanol and fractions with anti-*Pseudomonas* activity were collected and further purified by a semi-preparative HPLC column (ZORBAX SB-C18, 5 μm, 9.4 × 250 mm), using a mobile phase (40%MeOH:55%H_2_O:5%ACN) with a flow rate of 2.5 ml/min.

Purified mureidomycins were subjected to spectrometric analyses for structure determination. Electrospray Ionization-Mass Spectrometry (ESI-MS) and tandem Mass Spectrometry (MS/MS) analyses were carried out on Triple Quadrupole LC/MS system (Agilent 1260/6460). High-Resolution Mass Spectrometry (HR-MS) analysis was performed on Mass Quadrupole Time-of-Flight (Q-TOF) LC/MS system (Agilent 1200/6520). Nuclear Magnetic Resonance (NMR) spectra were recorded on a Bruker Advance spectrometer (AV500 MHz).

## Additional Information

**How to cite this article**: Jiang, L. *et al.* Identification of novel mureidomycin analogues via rational activation of a cryptic gene cluster in *Streptomyces roseosporus* NRRL 15998. *Sci. Rep.* **5**, 14111; doi: 10.1038/srep14111 (2015).

## Supplementary Material

Supplementary Information

## Figures and Tables

**Table 1:** ^1^H NMR and ^13^C NMR data for *N*-acetylmureidomycin E in D_2_O.

Position^a^		δ_C_(ppm)	δ_H_(ppm)	Extra signals due to conformers	
				δ_C_(ppm)	δ_H_(ppm)
Uracil-2	N-CO-N	151.0			
Uracil-4	CO-N	165.7			
Uracil-5	CH	102.2	5.37		
Uracil-6	CH	140.0	6.89	141.1	7.27
Sugar-1	O-CH-N	93.2	5.9		
Sugar-2	O-CH	72.5	4.29		
Sugar-3	CH_2_	33.0	2.77/2.55		
Sugar-4	>C=	144.2			
Sugar-5	−CH=	96.9	5.8		
AMBA-1	CO-N	167.7			
AMBA-2	CH	55.23	4.43	55.67	4.51
AMBA-3	CH	50.6	4.76		
AMBA-4	CH_3_	13.15	1.06		
AMBA-N-CH_3_	N-CH_3_	27.97	2.49	30	2.96
Met^SO^-1	CO-N	173.45			
Met^SO^-2	CH	52.5	4.19	52.8	4.19
Met^SO^-3	CH_2_	24.73	2.03/1.91		
Met^SO^-4	CH_2_	48.9	2.75		
Met^SO^-6	CH_3_	36.6	2.54		
*m*-Tyr-1	COOH	176.5			
*m*-Tyr-2	CH	54.3	4.32		
*m*-Tyr-3	CH_2_	37.1	2.94/2.79		
*m*-Tyr-1’	ArC	138.7			
*m*-Tyr-2’	ArCH	114.1	6.65		
*m*-Tyr-3’	Ar-C-O	155.7			
*m*-Tyr-4’	ArCH	116.0	6.59		
*m*-Tyr-5’	ArCH	130.1	7.1		
*m*-Tyr-6’	ArCH	121.3	6.67		
Ureido	N-CO-N	158.4			
*m*-Tyr-1	CO-N	173.2			
*m*-Tyr-2	CH	51.4	4.79		
*m*-Tyr-3	CH_2_	36.5	2.77		
*m*-Tyr-1’	ArC	138.2			
*m*-Tyr-2’	ArCH	113.8	6.65		
*m*-Tyr-3’	Ar-C-O	155.7			
*m*-Tyr-4’	ArCH	115.5	6.59		
*m*-Tyr-5’	ArCH	129.9	7.1		
*m*-Tyr-6’	ArCH	120.8	6.67		
*N*-acetyl-1	CO-C H_3_	173.41			
*N*-acetyl-2	CH_3_	21.32	1.76		

^a^Abbreviations for the structure units: *m*-Tyr (*m*-tyrosine), Met^SO^ (methionine sulfoxide), AMBA (2-amino-3-methylaminobutyric acid).
