# Supplementary material for: Identification of novel mureidomycin analogues via rational activation of a cryptic gene cluster in Streptomyces roseosporus NRRL 15998
Source: Sci Rep. 2015 Sep 15;5:14111. doi: 10.1038/srep14111 (PMC4572928; doi:10.1038/srep14111)
Supplement: Supplementary Information [file srep14111-s1.pdf]

Identification of novel mureidomycin analogues via rational activation of a cryptic gene cluster in *Streptomyces roseosporus* NRRL 15998

Lingjuan Jiang[a,b,#], Lu Wang[a,c,#], Jihui Zhang[a], Hao Liu[c], Bin Hong[d], Huarong Tan[a*], Guoqing Niu[a*]

[a] State Key Laboratory of Microbial Resources, Institute of Microbiology, Chinese Academy of Sciences, Beijing, China

[b] University of Chinese Academy of Sciences, Beijing, China

[c] Key Laboratory of Industrial Fermentation Microbiology, Ministry of Education, College of Biotechnology, Tianjin University of Science and Technology, Tianjin, China

[d] Key Laboratory of Biotechnology of Antibiotics of Ministry of Health, Institute of Medicinal Biotechnology, Chinese Academy of Medical Sciences and Peking Union Medical College, Beijing, China

[#] J.L. and W. L. contributed equally to this work.

[*] Address correspondence to Guoqing Niu, niugq@im.ac.cn, or Huarong Tan, tanhr@im.ac.cn State Key Laboratory of Microbial Resources, Institute of Microbiology, Chinese Academy of Sciences, Beijing 100101, China

Supplementary Information

Supplementary Tables

Table S1. Comparison of ORFs of *S. roseosporus* NRRL 15998 with genes from *Streptomyces* sp. DSM 5940

ORF[a]	aa	*nps*[b]	aa identity (%)	Proposed function[c]
02978	344	*A*	99	ArsR family transcriptional regulator
02979	157	*B*	97	N-acetyltransferase
02980	255	*C*	99	phosphatase
02981.a[*]	94	*P1*	99	NRPS T-domain
02981.b[*]	407	*P2*	96	NRPS C-domain
02982	78	*D*	97	MbtH-like protein
02983	466	*E*	98	FAD-depending oxidoreductase
02984	876	*P3*	97	NRPS A-T-domains
02985	121	*F*	97	cupin2 domain-containing protein
02986	556	*P4*	96	NRPS C-T-domains
02987	857	*P5*	98	NRPS A-T-TE-domains
02988	466	*G*	99	argininosuccinate lyase
02989	307	*H*	99	homoserine kinase
02990	755	*I*	99	Daba synthase
02991	347	*J*	99	threonine aldolase
02992	280	*K*	98	phenylalanine 3-hydroxylase
02993	184	*L*	95	hypothetical protein
02994	511	*P6*	98	NRPS A-domain
02995[**]	273	*M*	97	hypothetical protein
02996	350	*N*	97	hypothetical protein
02997	424	*O*	97	major facilitator superfamily transporter
02998	398	*P7*	99	NRPS C-domain

02999	430	*Q*	97	aminotransferase
03000	311	*R*	99	dioxygenase
03001	235	*S*	99	hypothetical protein
-		*T*		hypothetical protein
03002	327	*U*	94	FMN-depending oxidoreductase
03003	526	*P8*	97	NRPS A-domain
03004	237	*V*	99	methyltransferase

[a] ORFs of *S. roseosporus* NRRL 15998. [b] Genes from napsamycin gene cluster of *Streptomyces* sp. DSM5940. [c] Gene functions were proposed based on deduced or established functions in *Streptomyces* sp. DSM5940. [*] Two orfs in the *SSGG_02981*. [**] Revised *SSGG_02995*.

Table S2. Strains and plasmids used in this study

Strains/plasmids	Genotype or description	Reference or source
E. coli		
Top10	F⁻ *mcr*A Δ(*mrr-hsd*RMS-*mcr*BC) Φ80*lac*ZΔM15 Δ*lac*X74 *rec*A1 *ara*D139 Δ(*ara leu*) 7697 *gal*U *gal*K *rps*L (StrR) *end*A1 *nup*G	Invitrogen
ET12567	*dam dcm hsdS cat tet*	[1]
Pseudomonas		
P. aeruginosa PAO1	indicator strain	[2]
P. aeruginosa PA14	indicator strain	[3]
Streptomyces		
S. roseosporus NRRL 15998	daptomycin producer, wild-type strain	Broad Institute
Sros-02978D	A derivative of *S. roseosporus* NRRL 15998 with deletion of *SSGG-02978*	This study
Sros-h02995	A derivative of *S. roseosporus* NRRL 15998 containing containing pSET152::h02995	This study
Sros-A	A derivative of *S. roseosporus* NRRL 15998 containing pSET152::A	This study
Sros-hA	A derivative of *S. roseosporus* NRRL	This study

	15998 containing pSET152::hA	
Sros-02988D	A derivative of Sros-hA with disruption of *SSGG-02988*	This study
S. albus J1074	A derivative of *S. albus* G1 (DSM 41398) defective in the *Sal*GI restriction-modification system	[4]
S. coelicolor M1146	Δ*act* Δ*red* Δ*cpk* Δ*cda*	[5]
S. coelicolor M1152	Δ*act* Δ*red* Δ*cpk* Δ*cda rpoB(C1298T)*	[5]
S. coelicolor M1154	Δ*act* Δ*red* Δ*cpk* Δ*cda rpoB(C1298T) rpsL (A262G)*	[5]
J1074-mur	A derivative of *S. albus* J1074 containing the mureidomycin biosynthetic gene cluster	This study
M1146-mur	A derivative of *S. coelicolor* M1146 containing the mureidomycin biosynthetic gene cluster	This study
M1152-mur	A derivative of *S. coelicolor* M1152 containing the mureidomycin biosynthetic gene cluster	This study
M1154-mur	A derivative of *S. coelicolor* M1154 containing the mureidomycin biosynthetic gene cluster	This study

**Plasmids**		
pBluescript II KS+	Routine cloning vector	Stratagene
pUC119::*neo*	pUC119 containing kanamycin resistance gene (*neo*)	[6]
pUZ8002	*tra neo* RP4	[7]
pSET152	*oriT* RK2 *aac(3)IV int* ϕC31	[8]
pKC1139	*oriT* RK2 *aac(3)IV* rep^{ts}	[8]
pKC1139::*nap*	A derivation of pKC1139 containing the napsamycin/mureidomycin biosynthetic gene cluster from *S. roseosporus* NRRL 15998	[9]
pIJ10500	pMS82 derivative containing hygromycin resistance gene	[10]
pKC1139hph	pKC1139 derivative with the replacement of apramycin resistance gene by hygromycin resistance gene from pIJ10500	This study
pKC1139::02978UD	pKC1139 derivative with insertion of 2.0 kb fragments upstream and downstream of SSGG-02978 coding region	This study
pKC1139::02978UDneo	pKC1139::02978UD derivative with insertion of kanamycin resistance cassette	This study

	into the SpeI site	
pSET152::h02995	pSET152 derivative with insertion of *SSGG-02995* driven by *hrdB* promoter	This study
pSET152::A	pSET152 derivative with insertion of *ssaA* and its upstream region	This study
pSET152::hA	pSET152 derivative with insertion of *ssaA* driven by *hrdB* promoter	This study

Table S3. Primers used in this study

Primer	Sequence ($5' \rightarrow 3'$)[a]	Purpose
02978-UpF	AATTCTAGAGACTCATATCGGGAGCTCCTC	*SSGG-02978* deletion
02978-UpR	AATTACTAGTCAGAAAGTCCGGGTAATAAGC	*SSGG-02978* deletion
02978-DnF	AATTACTAGTGAGCTGACGGATCTGGTCGC	*SSGG-02978* deletion
02978-DnR	AATTGATATCACCACACGGCACACCCG	*SSGG-02978* deletion
Kan-F	AATTACTAGTTCCCCTGGATACCGCTCGC	*SSGG-02978* deletion
Kan-R	TTAAACTAGTACCCGAACCCCAGAGTCCC	*SSGG-02978* deletion
02995-F	ATGGACAACCTTTTCGAGCGTG	*SSGG-02995* overexpression
02995-R	AATAGCGGCCGCAGGAGAGTGACGAGCGCTTGGCG	*SSGG-02995* overexpression
ssaA-F	ATATCTAGACGCACCAGGAGCCCCGCCCG	*ssaA* overexpression
ssaAorf-F	ATGAATACCATGACCGAATCCG	*ssaA* overexpression
ssaA-R	AATAGCGGCCGCTCAGGCACATTGTGCCCTCG	*ssaA* overexpression
hrdBp-F	AATTTCTAGACCGCCTTCCGCCGGAACG	*SSGG-02995* and *ssaA* overexpression
hrdBp-R	GAACAACCTCTCGGAACGTTGA	*SSGG-02995* and *ssaA* overexpression
02983-SCF	GTGACAGTGGGCCCGGCGGT	*SSGG-02983* disruption
02983-SCR	GGGCCGCCGTCGAACGACAC	*SSGG-02983* disruption

02988-SCF	ATCACGCCGGGCTTCTACCTC	*SSGG-02988* disruption
02988-SCR	AACAGGTTGCTCGCGTGCAG	*SSGG-02988* disruption
02979-RTF	AGTTGTTCTCCTCGCCGGAGG	RT-PCR analysis
02979-RTR	ATTCCTCGAACCGCTCCACC	RT-PCR analysis
02987-RTF	GCCTATCTGCCGATTGAT	RT-PCR analysis
02987-RTR	GACCTCGTGTGTCGTGTC	RT-PCR analysis
02992-RTF	ATGCAGAGGAAGCACGCCTGC	RT-PCR analysis
02992-RTR	CGAGCTCGCCGTACGAGGAGA	RT-PCR analysis
02997-RTF	GCGAACGATTCTCAGGGTGTCG	RT-PCR analysis
02997-RTR	GACTGAGTGGTGCCGAGCTT	RT-PCR analysis
hrdB-RTF	ACCAGATTCCGCCAACCC	RT-PCR analysis
hrdB-RTR	CTCCTCTTCCTCGCCCTT	RT-PCR analysis

[a] Underlined, restriction enzyme recognition sites.

Supplementary Figures

Coding sequence of amended *SSGG_02995*

ATGGACAACCTTTTCGAGCGTGATGCAGAAGTGATT**CTG**ACCTGGCGCGTCCAGGCCCGCCACACGGGTGGATCCACTACCCAGGAAAGGATCAACCT
GAAAGTCGGCGATCGACTGGACTTCGGCATACCCAACGCCACCGGAGAATTCCAGCCCAATGCTGCGCCCAGCATCGGCACCTTTGAAGTGTCCGACC
AGCACCTCTTCTTCTCGAACTTCACCACCGGCACCACGTTCGTCATCGAGAATTTGGAGGGTGGGACTGAACTCGTCAAGGCGCGCCCCCGCCAGCTC
GGCATGGTGATCCCGTTCGAGATGTCGCGCATCCTCATCCCCTCCAGCGCAAGCATCGGCGAACTCACCGTCTTCACATCCCCGCCCCGCCTGCTCGG
GCCCGAAATGGCCGCCACGCCTACCGAGTCCGCGATGTCCAAACTGGACACGACGACCAAGTACTTCGCCGTTCTTGTCGCCTTGTGCGAGCCGCGTC
TGCGCGGTTCCTCGATGGCGGCCGTGCCCTCCGTCCAAGAGGTGGTCGAAAGACTGCGGGGATCGAGCAGTTCAGGGACGCCAACCGCAGCAGCGTC
AATTACCACATCGACTATCTGGCTGACCAGAAGCTTCCGGTCAGCCAGTGGGCCAAGTACCTGAACGAAGGGCGCATGCATTCGAAGCGCGAGGCCCT
GGTCGCCTTCTCGCTCCGCTTCGATCTGGTCCGCGAGGAACACCTCTCTCTCCTCCCCGACGTCAAACTCCTGCGCGCCCAACGATGTCTTGAGGCAC
GGCAAGCCACGAAGGTGGCAACGGGCGCGATCTCATGA

Coding sequence of amended *ssaA*

ATGAATACCATGACCGAATCCGATGTAGAA**GTG**ATCCTGACCTGGCGGAACAAGACCCGCCACCACGGCAGCTCCGACGCCAAGGAACGGATCAACCT
GAAGGTCGGCGAACGCCTGAACTTCGGCATATCGAGCTCCACCGGCGAATTCCAGCCCGGCGCCCTGCCGAGCATCGGAACGTTCGAAGTGTCCGACC
ATCACCTGCTCTTCTCGAACTTCACGACGAGCACCACGTTCGTCATCGAGAACCTGGAGGGCGGCACCGAACTGGTCAAGGCCCGCCCGCGTCAGCTG
GGCATGGTGATCCCGTTCGAGATGTCGCGCATCCTGATCCCGTCCAGCGAGACCATCAGCGAACTGACGGTCTTCACTCCCCCTCCGCGCCTGCTCGG
CTCCGACCAGGCCGCCACCCTCACCGAGTCCGCGATGTCCAAGCTGGACACGGCGAGCAAGTACTTCGCCGTCCTCGTGGCCCTGTGCGAGCCGCGAC
TGCGCGGTTCCTCCATGGCGGCCGTCCCCTCCGTGCAGGAGGTGGTCGAACGGCTGCGCGGGATCAAGCAGTTCAAGGGCGCGAACCGCAGCAGCATC
AACTACCACATCGACTACCTGGCGGATCAGAAGCTTCCGGTCGGCCAGTGGGCCAAGTACGTGGACGAGGGCCGCATGCACTCCAAGCGCGAGGCCCT
GGTCGCGTTCTCGCTCCGGTTCGACCTGGTCCGGGAGGAACACCTGGCGCTGCTTCCCGATCTCAAACGTGCGAGGGCACAATGTGCCTGA

Coding sequence of *SSGG_02981.a*

ATGAGTCTGCAGAACGCAAAGGCGCCGGAACGCGTCGCGGTCCTGACCCGGATCTGGCGAGAGGTTCTCGGAGATCCCGCCCTCGACGAGGATGCGGA
TCTCTTCGAGAAGAACGGATCGTCGTTGCAGGTCCTGCAGATCACCGGCAGGATCCACGACGCCCTCGGTATCGACGTGAAGCTGCGTCACGTATTCT
CGTACGCCTCGCCGCGCAGCCTTTCCGATTTCCTCGACGCCGAGTTCGCGCAGGGGGATTCCGGCGCGGTGCACGGCGGCGAGAAGTGA

Coding sequence of *SSGG_02981.b*

ATGGCCCGTCTGACCGGCGAGTTCGCCGATTGGCACAACCTTCACTACATGGCGATGTGGCTCACCGGCGAACTGGACGTCGCCGCCCTCCGCGACGC
GTGGTGGCGGGTGTGCCTCCGGCACGACGCCCTGCGCCGCACCTATCTCTCGCCGGAGGAGGCGCGCACCGATGGAGACGCGCTCAGCGAGGTCGAGT
TCCTGACCGCCGCGTCGGACGCGGACGCGGTCGAGCTGATGAGCCGCTTCATCGGCCGCCCCTTCGACCTGGGCGGCCCGAGCTTCTCACGGGTCGCC
GTCGTGCGGTGCGGCGAGCGCAGGCACCTGTTCGGGATCGCGATCGACCACATCATCAACGACCTGGCCTCGTGGGCCCTCATCCGGGCGGACTTCAC
CGAGTTCTACCGCCGCGCCCTGACCGGCGATTCCGGCGATGTCACGGCCGCCGGCAGCTACCAGAGCTTCGCCTCCGAGGAGCGGCGTCTGTTCGCCG
GCCGGTGGGGGCAGGAGTGCCGGGCGTTCTGGCGCTCCTACGTCGACGAGTTCGGCACGGTCCCCGCGCCCTTCCCGGTCGGCGGCGACACCACGGGC
GCTTACCGGCCCAGGACGATCAGCCGTGTGCTGCCTGCCGACGCGAAGGCGCGCCTGCACGCGCTCTCGCTGGAGGCCCGGGCGACACCCTTCGCCGC
GGTCACCGCCGGCGTGCTCGCCGCCGCGAAGGAGGTCACCGGCGATCCGGTGGCCGGAATCTCCGTCAACCAGCACGGGCGGATGCTGCCCGGCACGT
CGCAGACCGCCGGGCTGTTCGTCCAGACTGTGCCGCTGCACCTCGGGGCACCGCACAAGAGCCCGGCCGAGGCGGTCCGGGAGGTCTTCCACCGGACC
CACGACGTCTTCGAGTACAGCGTTCCGCTGCTGGTCGCCGGACGGTACTGGAGCGAGACCCTGATGGTGCCGGACCGGCAGGCCGGGCTGTACGTCAG
TCTCAACGAGGAGCCGCCCTCCTCGCACGACCTGCCGCCGTTCGCCGGCACCGAGGCGGAGTACGTCGAACTCGACGTCCCCGGCGGGAAACGGTTCC
CGGAAACCGTGGTCGTCGCGTGGAACCTGTACGAGACGGGCCCGGAGCTCGTCGCGCACTACAACGAGGACCGCTTCCCCGGCGAGGCCGTGGAACAG
CTCCTGGAAGCGGCCGAGCGGTTCGTACTCCCCGCAGACGCACAGTGA

Figure S1. Nucleotide sequences of *SSGG-02981.a*, *SSGG-02981.b*, and amended nucleotide sequences of *SSGG-02995* and *ssaA*.

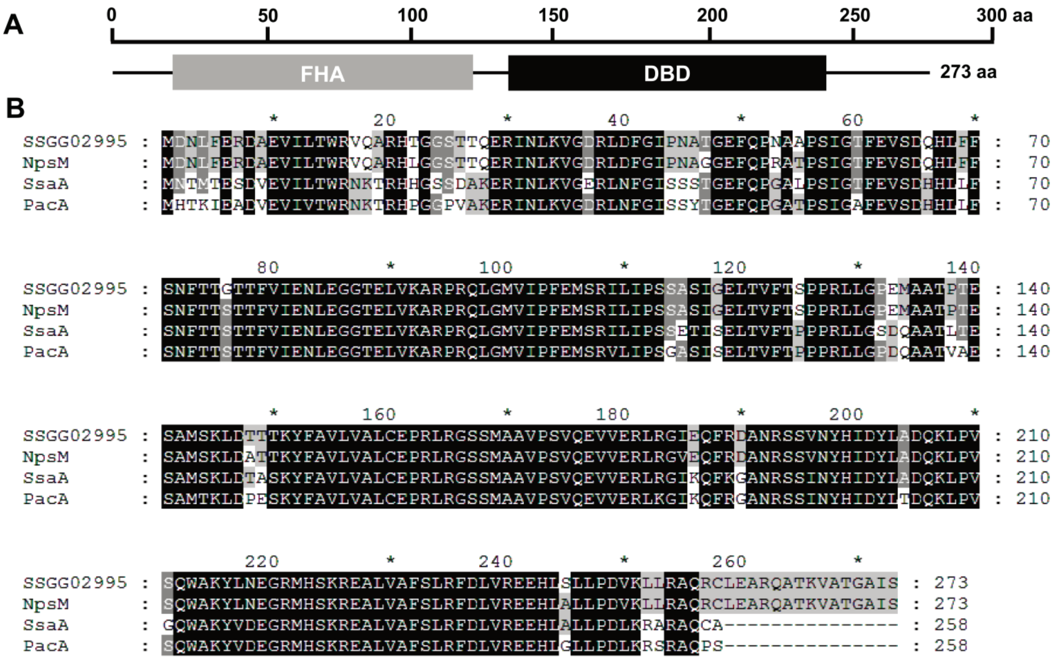

Figure S2. Domain structure and amino acid alignment of SSGG02995 protein. (A) Predicted domain structure of SSGG02995. FHA: N-terminal fork head-associated domain; DBD: C-terminal LuxR-type helix-turn-helix (HTH) DNA binding domain. (B) Alignment of SSGG02995 with homologous proteins from other *Streptomyces* species. SSGG02995, a hypothetical protein from *S. roseosporus* NRRL 15998 (GenBank accession number ZP_04694256.1); NpsM, a hypothetical protein from *Streptomyces* sp. DSM 5940 (GenBank accession number ADY76675.1); SsaA, a regulator from *Streptomyces* sp. SS (GenBank accession number AGG82463.1); PacA, a hypothetical protein from *S. coeruleorubidus* (GenBank accession number ADN26237.1). Identical or positive amino acid residues are shown in black, and similar residues are shaded.

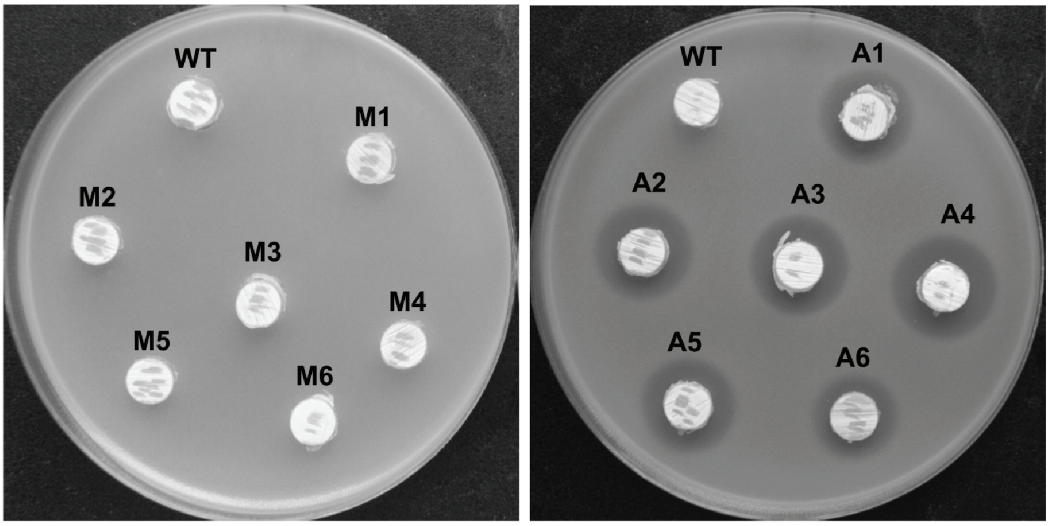

Figure S3. Bioassay of mureidomycin production against *P. aeruginosa* PAO1. *S. roseosporus* NRRL 15998 (WT) and its derivatives were cultured on ISP-2 agar for 4 days, and agar plugs were assayed for bioactivity against *P. aeruginosa* PAO1. WT, plugs from the wild-type strain. A1-A6, plugs from six different colonies of Sros-hA. M1-M6, plugs from six different colonies of Sros-h02995.

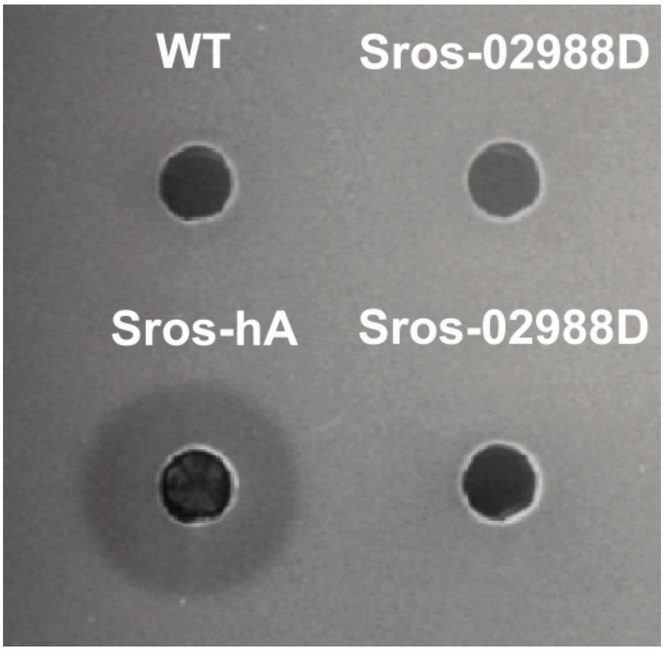

Figure S4. Bioassay of mureidomycin production in *S. roseosporus* NRRL 15998 (WT), Sros-hA and *SSGG-02988* disruption mutants (Sros-02988D). The stains were cultured in liquid ISP-2 for 6 days, and 100 µl of supernatants from culture broths were assayed for bioactivity against *P. aeruginosa* PA14.

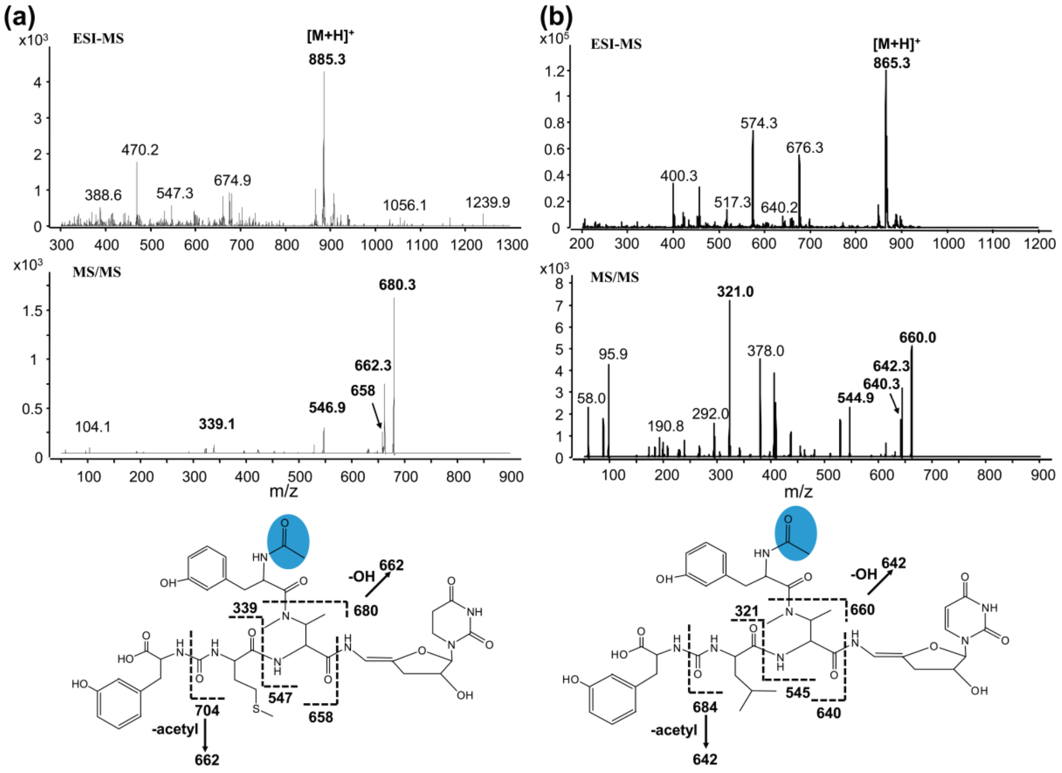

Compound **2** (*N*-acetylmureidomycin B) Compound **6** (*N*-acetylmureidomycin H)

Figure S5. ESI-MS and MS/MS analyses of compound 2 (*N*-acetylmureidomycin B) and compound 6 (*N*-acetylmureidomycin H). The leucine in *N*-acetylmureidomycin H could also be an isoleucine. The unique acetyl group was shadowed with an oval.

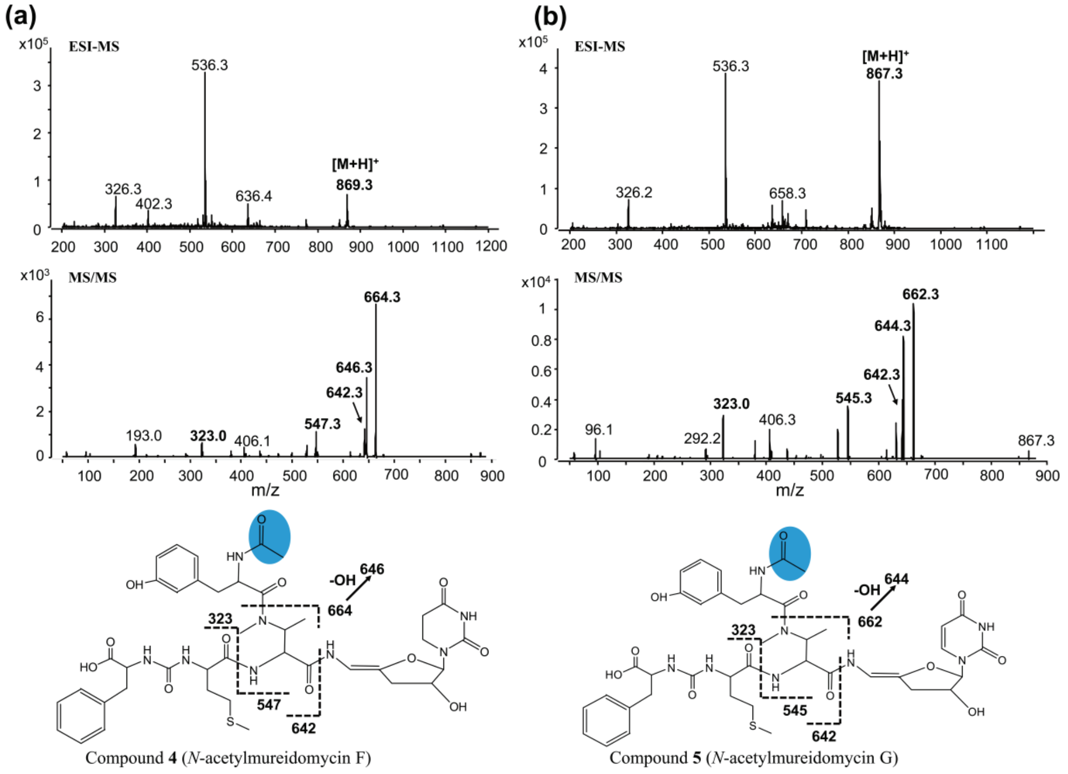

Figure S6. ESI-MS and MS/MS analyses of compound 4 (*N*-acetylmureidomycin F) and compound 5 (*N*-acetylmureidomycin G). The unique acetyl group was shadowed with an oval.

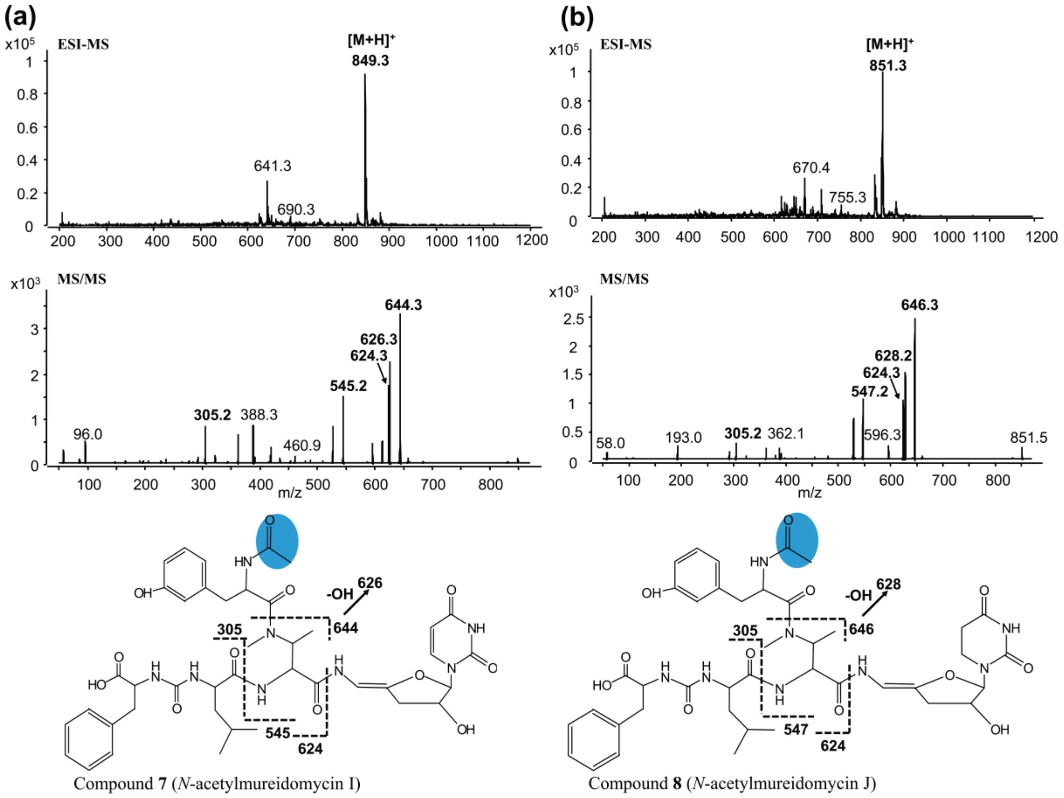

Figure S7. ESI-MS and MS/MS analyses of compound 7 (*N*-acetylmureidomycin I) and compound 8 (*N*-acetylmureidomycin J). The leucine in *N*-acetylmureidomycin I and J could also be an isoleucine. The unique acetyl group was shadowed with an oval.

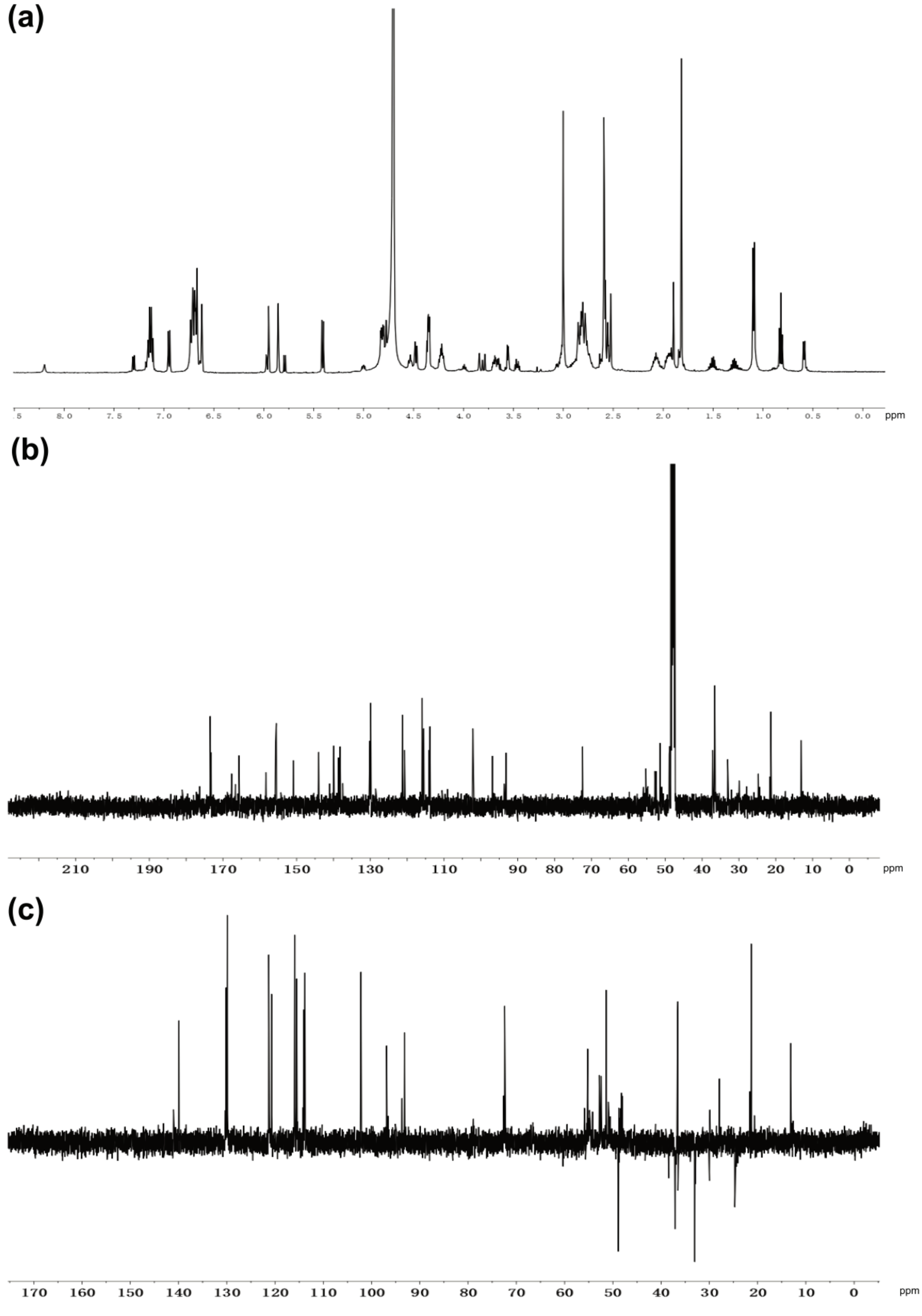

Figure S8. ^{1}H-NMR spectrum (A), ^{13}C-NMR spectrum (B) and DEPT-135 spectrum (C) of compound 1 (*N*-acetylmureidomycin E).

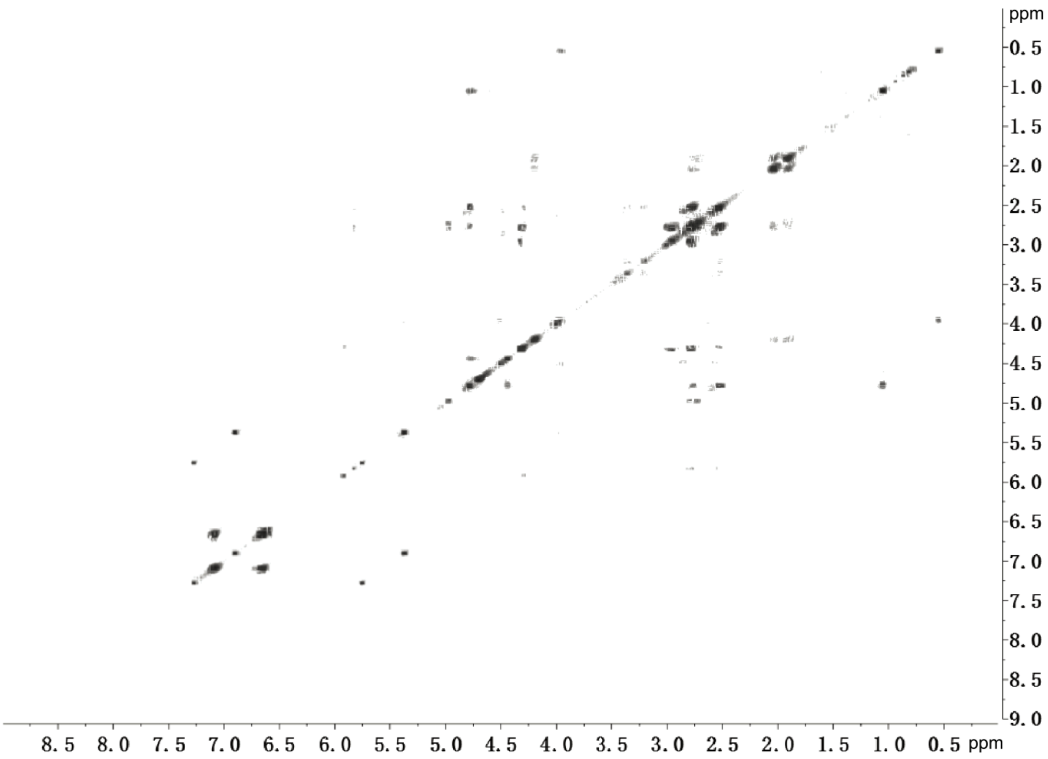

Figure S9. COSY spectrum of compound 1 (*N*-acetylmureidomycin E).

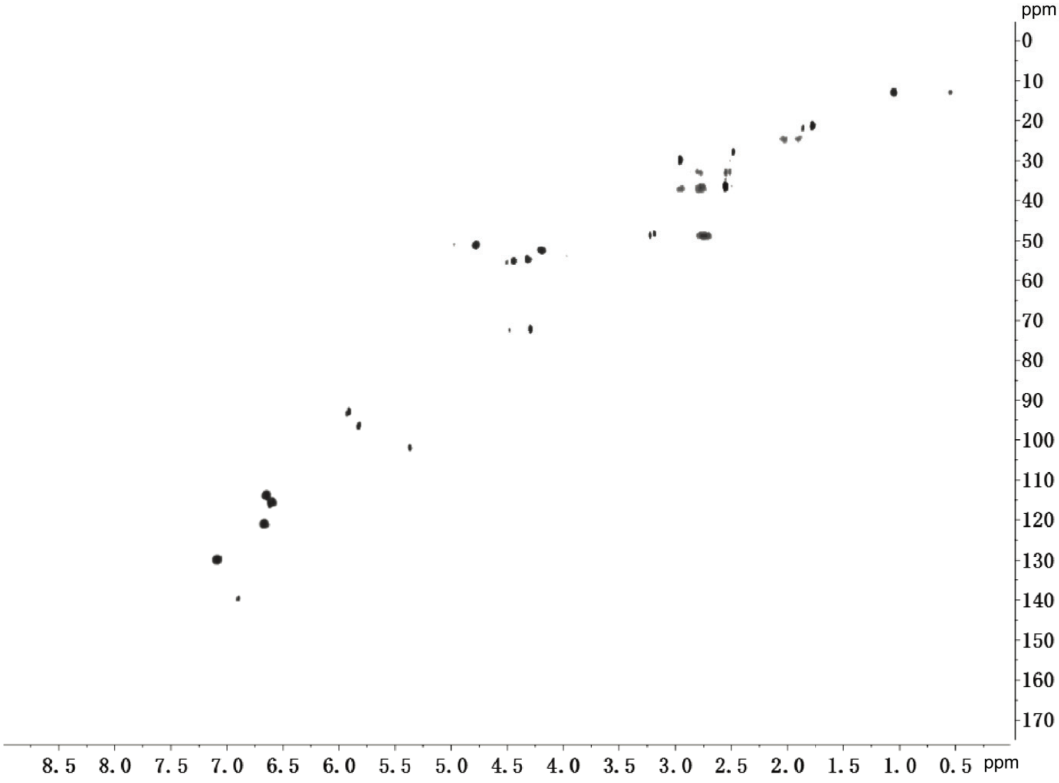

Figure S10. ^{1}H-^{13}C HSQC spectrum of compound 1 (*N*-acetylmureidomycin E).

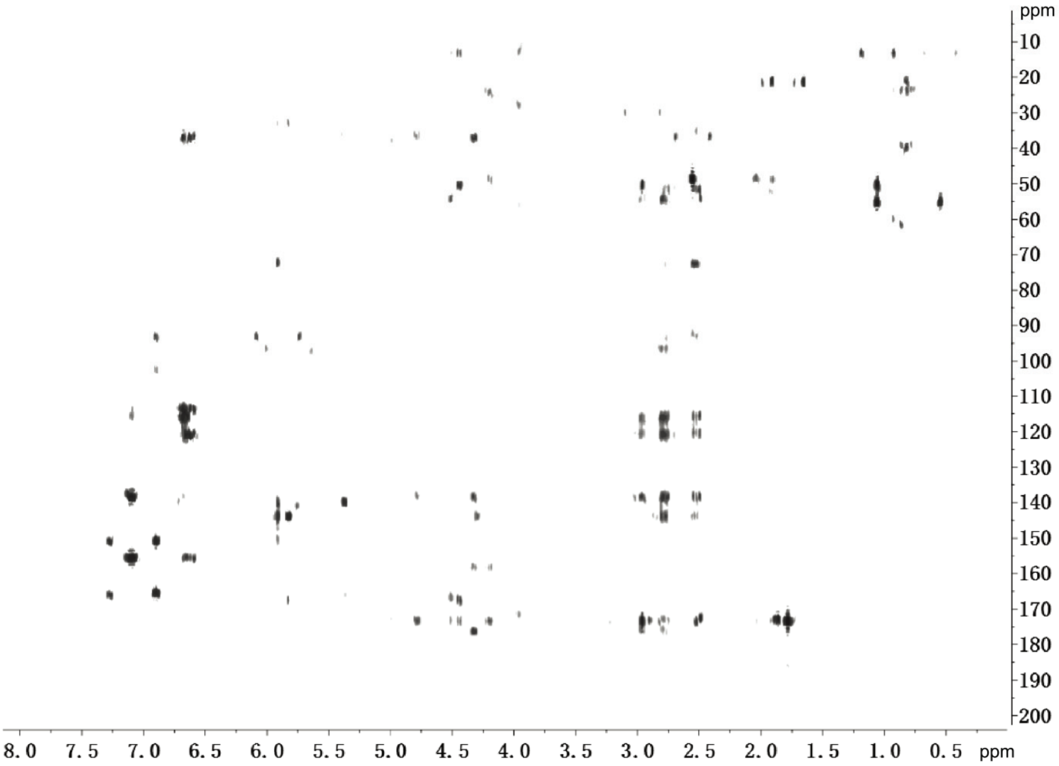

Figure S11. HMBC spectrum of compound 1 (*N*-acetylmureidomycin E).

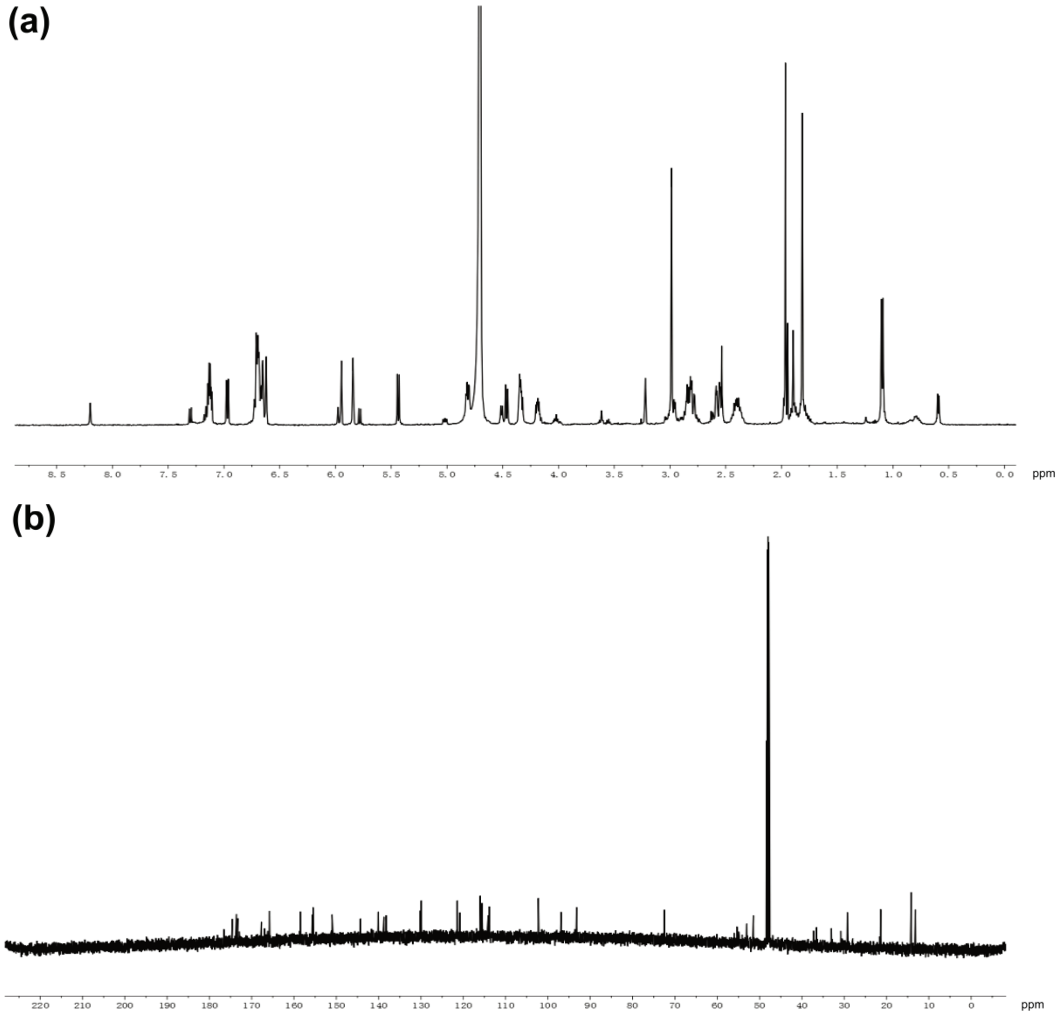

Figure S12. ^{1}H-NMR spectrum (A) and ^{13}C-NMR spectrum (B) of compound 3 (*N*-acetylmureidomycin A).

References

1. MacNeil, D. J. *et al.* Analysis of *Streptomyces avermitilis* genes required for avermectin biosynthesis utilizing a novel integration vector. *Gene* **111**, 61-68 (1992).
2. Stover, C. K. *et al.* Complete genome sequence of *Pseudomonas aeruginosa* PAO1, an opportunistic pathogen. *Nature* **406**, 959-964 (2000).
3. Lee, D. *et al.* Genomic analysis reveals that *Pseudomonas aeruginosa* virulence is combinatorial. *Genome Biol.* **7**, R90 (2006).
4. Chater, K. F. & Wilde, L. C. *Streptomyces albus* G mutants defective in the *Sal*GI restriction-modification system. *J. Gen. Microbiol.* **116**, 323-334 (1980).
5. Gomez-Escribano, J. P. & Bibb, M. J. Engineering *Streptomyces coelicolor* for heterologous expression of secondary metabolite gene clusters. *Microb. Biotechnol.* **4**, 207-215 (2011).
6. Li, R. *et al. polR*, a pathway-specific transcriptional regulatory gene, positively controls polyoxin biosynthesis in *Streptomyces cacaoi* subsp. *asoensis*. *Microbiology* **155**, 1819-1831 (2009).
7. Paget, M. S., Chamberlin, L., Atrih, A., Foster, S. J. & Buttner, M. J. Evidence that the extracytoplasmic function sigma factor σ^E is required for normal cell wall structure in *Streptomyces coelicolor* A3(2). *J.Bacteriol.* **181**, 204-211 (1999).
8. Bierman, M. *et al.* Plasmid cloning vectors for the conjugal transfer of DNA from *Escherichia coli* to *Streptomyces* spp. *Gene* **116**, 43-49 (1992).
9. Du, D. *et al.* Genome engineering and direct cloning of antibiotic gene clusters via phage ϕBT1 integrase-mediated site-specific recombination in *Streptomyces*. *Sci. Rep.* **5**, 8740

(2015).

10. Pullan, S. T., Chandra, G., Bibb, M. J. & Merrick, M. Genome-wide analysis of the role of GlnR in *Streptomyces venezuelae* provides new insights into global nitrogen regulation in actinomycetes. *BMC Genomics* **12**, 175 (2011).
